# Double-J placement complicated by endogenous endophthalmitis: A rare and serious outcome

**DOI:** 10.1016/j.eucr.2023.102608

**Published:** 2023-11-02

**Authors:** Omar Bellouki, Amine Essakali, Imad Boualaoui, Ahmed Ibrahimi, Hachem El Sayegh, Yassine Nouini

**Affiliations:** a Urology “A” Department, Ibn Sina University Hospital of Rabat, Morocco; b Ophthalmology “B” Department, Ibn Sina University Hospital of Rabat, Morocco; c Faculty of Medicine and Pharmacy of Rabat, Mohammed V University, Rabat, Morocco

**Keywords:** Double-J stent, Endogenous endophthalmitis, Bacteremia, Intrarenal pressure

## Abstract

Endogenous endophthalmitis (EE) is a rare intraocular infection resulting from hematogenous microorganism spread, typically associated with underlying risk factors. We present a 52-year-old female with poorly-controlled diabetes and urolithiasis who developed EE following double-J stent placement for obstructive pyelonephritis. EE may occur due to increased intrarenal pressure during urological procedures. Timely recognition and intervention for rare complications like EE in urological patients are essential. This case highlights the importance of vigilance and collaboration between urologists and ophthalmologists in managing such cases.

## 1 Introduction

Endogenous endophthalmitis, marked by severe intraocular infection and the risk of blindness, arises when microorganisms travel through the bloodstream from a distant infectious source.^1^ While it is often associated with immunosuppression, less commonly, it can be provoked by surgical procedures.^2^ This case presents a unique scenario: EE developing after the placement of a double-J stent in a patient with obstructive pyelonephritis, possibly attributable to intrarenal pressure fluctuations.

## 2 Case presentation

A 52-year-old female with poorly-controlled type-2 diabetes and recurrent urolithiasis presented to our emergency department with a three-day history of right flank pain, fever, and oliguria. Upon examination, the patient had a fever of 39.5 °C, stable vital signs, and right lumbar tenderness, along with normal abdominal tone and the presence of an uncomplicated linea alba hernia.

**Fig. 1:**
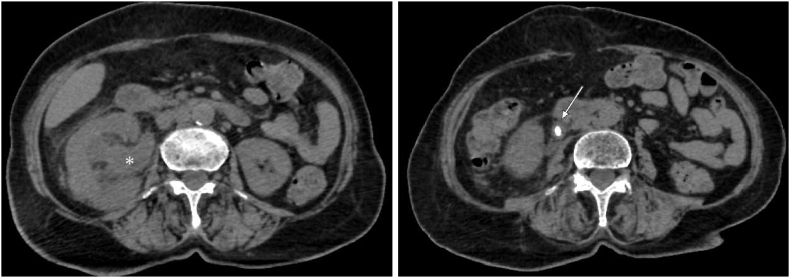
Non-contrast AP CT scan showing the ureteral stone (*arrow*) and the associated hydronephrosis (*asterisk*).

Laboratory tests revealed hyperleukocytosis at 19,000/μL, a CRP level of 325 mg/L, a creatinine level of 90 μmol/L, and an elevated HbA1c level of 10 %. Blood cultures were obtained. A non-contrast abdominal-pelvic CT scan revealed right-sided hydronephrosis caused by an 11 mm ureteral stone (Fig. 1).

The patient received intravenous ceftriaxone (1 g) in combination with a single-dose amikacin (30 mg/kg) prior to undergoing a ureteral double-J stent placement. The procedure was challenging as advancing the guidewire through the calculus zone necessitated significant manipulation and the use of retrograde pyelography. A perioperative hemodynamic instability occurred, but was effectively managed. Postoperatively, the patient showed favorable clinicobiological outcomes and was switched to oral ciprofloxacin, 500 mg twice daily, as urine culture confirmed the presence of fluoroquinolone-susceptible *Klebsiella pneumoniae*. Blood-culture remained negative.

**Fig. 2:**
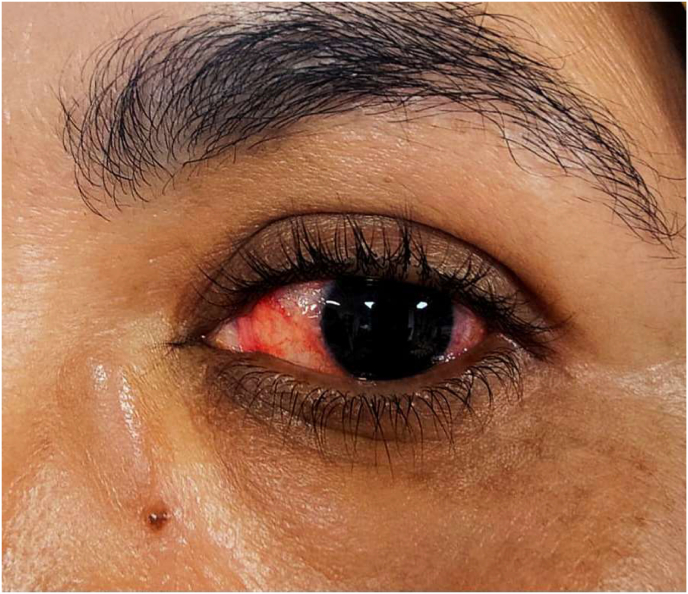
Clinical presentation of the patient's left eye, demonstrating conjunctival hyperemia with clear cornea.

**Fig. 3:**
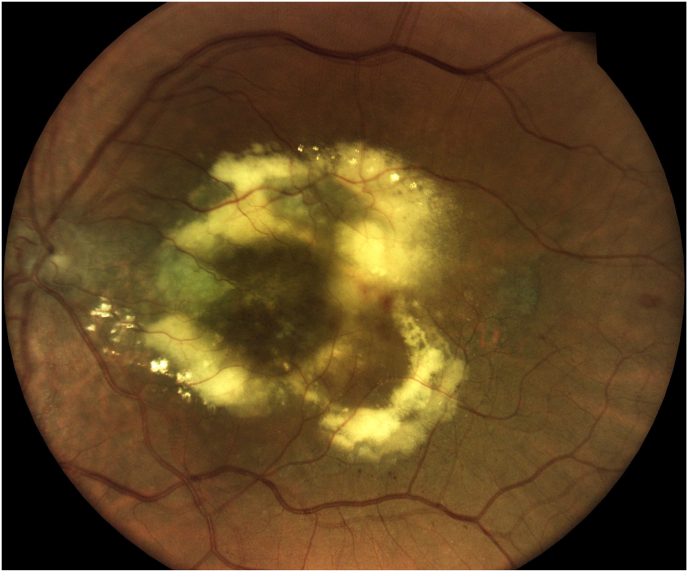
Fundus examination of the patient's left eye revealing multifocal yellow-white chorioretinal lesions.

An unusual incident occurred on postoperative day 3 when the patient reported pain, redness and progressive vision decrease in the left eye, along with a central scotoma (Fig. 2). An urgent ophthalmologic examination revealed reduced visual acuity of 1/10, normal intraocular pressure, and a normal anterior segment. Fundus examination showed central retinitis lesions with absence of foveal reflex (Fig. 3). Ocular ultrasound findings were consistent with the diagnosis of endophthalmitis.

The diagnosis of endogenous endophthalmitis was established. Our medical team supposed that this complication might be induced by a bacteremic discharge resulting from intrarenal pressure increase during the placement of the double-J stent, considering the perioperative hemodynamic instability. A transthoracic echocardiography found no signs of infectious endocarditis.

The patient underwent a vitrectomy along with intravitreal injection (IVI) of vancomycin (1 mg/0.1 mL) and ceftazidime (2.25 mg/0.1 mL), with culture samples obtained. IVI was repeated after 48 hours. The postoperative course was favorable, with reduced pain and improved visual acuity (4/10 with correction). Culture findings confirmed the presence of multi-sensitive *Klebsiella pneumoniae*, and oral ciprofloxacin was continued, along with topical steroids and fortified tobramycin.

## 3 Discussion

Endophthalmitis, a severe infection of the eye's inner layers, can result in vision impairment if not promptly treated.^1^ It occurs in different forms, including exogenous endophthalmitis (post-operative, post-traumatic, etc.) and endogenous endophthalmitis (EE), which accounts for 2–8% of all cases. EE is caused by hematogenous dissemination of microorganisms from a primary septic focus, commonly associated with bacteremia or fungemia.^2^ Gram-negative agents, such as Klebsiella, are frequently responsible for endogenous bacterial endophthalmitis (EBE).^3^

Many risk factors are associated with EE, including long-term indwelling catheters, intravenous drug abuse, chronic immunosuppression (e.g., cancer, AIDS, organ transplants), diabetes mellitus, infectious endocarditis, urinary tract infections, liver abscess, and recent surgical procedures.^2^^,^^4^ To the best of our knowledge, no cases of EBE triggered by ureteral double-J stent placement have been documented in the literature.

The pathophysiology of EBE involves the metastatic septic embolus spread of microorganisms from a primary infectious site into the eye. This dissemination is facilitated by bacteremia, enabling microbial proliferation and inflammatory reactions upon crossing the blood-ocular barrier. During endourological procedures, increased intrarenal pressure may cause the absorption of endotoxins, potentially leading to bacteremia.^2^^,^^5^

Clinical presentation of EE encompasses symptoms such as lid swelling, pain, redness, discharge, and decreased vision. Ophthalmologic assessments can reveal characteristic signs, such as vitreous opacities, hypopyon, corneal edema, and retinal alterations.^2^^,^^4^ Microbiological examination of blood and ocular fluids is essential to identify causative microorganisms.^1^

The management of EE begins with identifying and treating the infectious source. Local ophthalmological treatment for EE typically involves vitrectomy and intravitreal antibiotics, such as ceftazidime or amikacin, tailored to culture results. Systemic antibiotic therapy may be initiated using fluoroquinolones, commonly chosen for their effective vitreous penetration and broad-spectrum coverage.^2^^,^^4^

Although some degree of vision loss is common in EE, preserving any remaining vision is paramount. Despite adequate management, endophthalmitis may progress to fulminant forms, leading to panophthalmitis, ultimately requiring evisceration. The mortality rate for EBE stands at 38 %.^1^^,^^4^

In patients with risk factors, preventing such complications during endourological procedures involves using appropriately sized materials, controlling irrigation flow rates, avoiding the use of retrograde pyelography in infected patients, and monitoring patient hemodynamics.^2^^,^^5^

## 4 Conclusion

Although EE following double-J stent placement is uncommon, it serves as a reminder for urologists and healthcare providers to remain vigilant in their daily practice. Any untoward ocular symptoms following urological procedures warrant immediate ophthalmological consultation. Prompt recognition and effective management of this rare complication are vital not only for preserving the patient's vision but also for their overall well-being.

## Funding

No sources of funding are to be declared.

## Ethical approval

Ethical approval is not required by our institution.

## Consent

Written informed consent was obtained from the patient for publication of this case report and accompanying images.

## Declaration of competing interest

No conflicts of interests are to be declared by the authors.
